# Supplementary material for: Chest X-ray Does Not Predict the Risk of Endotracheal Intubation and Escalation of Treatment in COVID-19 Patients Requiring Noninvasive Respiratory Support
Source: J Clin Med. 2022 Mar 16;11(6):1636. doi: 10.3390/jcm11061636 (PMC8950017; doi:10.3390/jcm11061636)
Supplement: Supplementary file 1 [file jcm-11-01636-s001.zip › Table S1.pdf]

Table S1. Baseline and outcome variables

Baseline variables collected at NIRS initiation	• Age (years)<br>• Weight (kg)<br>• Body mass index (kg/m^2)<br>• Female gender (n [%])<br>• Hypertension (n [%])<br>• Obesity (n [%])<br>• Diabetes (n [%])<br>• Days since symptoms onset<br>• Sequential organ failure assessment score<br>• Charlson comorbidity index<br>• C-reactive protein (mg/L)<br>• Procalcitonin (μg/L)<br>• D-dimer (μg/L)<br>• Leukocyte count (x10^9 cells/L)<br>• Lymphocyte count (x10^9 cells/L)<br>• Interleukin-6 (pg/mL)<br>• Arterial partial pressure of oxygen to inspired oxygen fraction ratio (mmHg)<br>• Arterial partial pressure of carbon dioxide (mmHg)
Outcome variables	• Escalation of respiratory support (n [%])<br>• Pronation (n [%])<br>• Endotracheal intubation (n [%])<br>• Duration of invasive mechanical ventilation (days)<br>• Hospital length of stay (days)

	• Hospital mortality (n [%])
Abbreviations: NIRS, noninvasive respiratory support.	
