# Supplementary material for: Chest X-ray Does Not Predict the Risk of Endotracheal Intubation and Escalation of Treatment in COVID-19 Patients Requiring Noninvasive Respiratory Support
Source: J Clin Med. 2022 Mar 16;11(6):1636. doi: 10.3390/jcm11061636 (PMC8950017; doi:10.3390/jcm11061636)
Supplement: Supplementary file 1 [file jcm-11-01636-s001.zip › Table S3.pdf]

Table S3. Logistic regression for the escalation of respiratory support

Variable	Univariable		Multivariable	
	OR (95% CI)	p-value	OR (95% CI)	p-value
First CARE score	1.01 (0.96-1.07)	0.66		
Age	0.99 (0.96-1.02)	0.62		
Female gender	1.10 (0.91-1.37)	0.15		
Days since symptoms onset	1.07 (0.97-1.17)	0.16		
SOFA score	1.30 (0.98-1.71)	0.06		
Charlson comorbidity index	0.93 (0.81-1.06)	0.28		
C-reactive protein	1.01 (1.00-1.01)	0.05		
Procalcitonin	1.18 (0.91-1.53)	0.22		
D-dimer	1.00 (1.00-1.00)	0.44		
Leukocyte count	1.08 (1.01-1.17)	0.03	1.06 (0.98-1.15)	0.17
Lymphocyte count	0.91 (0.75-1.12)	0.38		
IL-6	1.00 (1.00-1.01)	0.18		
PaO2/FiO2	0.99 (0.99-1.00)	< 0.01	0.99 (0.99-1.00)	< 0.01
PaCO2	1.05 (0.99-1.11)	0.14		

Abbreviations: OR, odds ratio; CI, confidence interval; SOFA, sequential organ failure assessment; IL6, interleukin-6; PaO2/FiO2, arterial partial pressure of oxygen to inspired oxygen fraction ratio; PaCO2, arterial partial pressure of carbon dioxide.
