# Supplementary material for: Chest X-ray Does Not Predict the Risk of Endotracheal Intubation and Escalation of Treatment in COVID-19 Patients Requiring Noninvasive Respiratory Support
Source: J Clin Med. 2022 Mar 16;11(6):1636. doi: 10.3390/jcm11061636 (PMC8950017; doi:10.3390/jcm11061636)
Supplement: Supplementary file 1 [file jcm-11-01636-s001.zip › Table S4.pdf]

Table S4. Linear regression for endotracheal intubation duration

Variable	Univariable	
	Estimate (95% CI)	p-value
First CARE score	0.13 (-0.13-0.38)	0.32
Age	-0.11 (-0.28-0.07)	0.23
Female gender	-3.41 (-7.59-0.78)	0.11
Days since symptoms onset	0.42 (-0.14-0.98)	0.14
SOFA score	1.66 (0.16-3.16)	0.03
Charlson comorbidity index	-0.53 (-1.28-0.22)	0.16
C-reactive protein	0.01 (-0.02-0.04)	0.43
Procalcitonin	0.60 (-0.61-1.80)	0.33
D-dimer	-0.00 (-0.00-0.00)	0.30
Leukocyte count	0.05 (-0.36-0.47)	0.80
Lymphocyte count	-1.45 (-3.18-0.28)	0.10
IL-6	0.00 (-0.01-0.01)	0.96
PaO2/FiO2	-0.01 (-0.04-0.02)	0.46
PaCO2	-0.07 (-0.37-0.24)	0.67
Abbreviations: OR, odds ratio; CI, confidence interval; SOFA, sequential organ failure assessment; IL6, interleukin-6; PaO2/FiO2, arterial partial pressure of oxygen to inspired		

oxygen fraction ratio; $PaCO_2$, arterial partial pressure of carbon dioxide.
