# Supplementary material for: Chest X-ray Does Not Predict the Risk of Endotracheal Intubation and Escalation of Treatment in COVID-19 Patients Requiring Noninvasive Respiratory Support
Source: J Clin Med. 2022 Mar 16;11(6):1636. doi: 10.3390/jcm11061636 (PMC8950017; doi:10.3390/jcm11061636)
Supplement: Supplementary file 1 [file jcm-11-01636-s001.zip › Table S5.pdf]

Table S5. Comparison between hospital survivors and non-survivors

Variable	All patients (n = 142)	Survivors (n = 122)	Non-survivors (n = 20)	p-value
Age (years)	69 (58-75)	68 (57-74)	73 (70-80)	<0.01
Weight (kg)	78 (69-97)	77 (67-102)	79 (72-95)	0.45
Body mass index (kg/m^2)	26 (22-31)	25 (21-31)	27 (22-32)	0.33
Female gender (n [%])	44 (31)	39 (32)	4 (20)	0.31
Hypertension (n [%])	81 (57)	68 (56)	13 (65)	0.45
Obesity (n [%])	45 (32)	39 (32)	5 (25)	0.79
Diabetes (n [%])	38 (27)	33 (27)	5 (25)	1
Days since symptoms onset	6 (4-9)	6 (4-9)	6 (4-10)	0.96
SOFA score	3 (2-4)	3 (2-4)	4 (3-5)	<0.01
Charlson comorbidity index	3 (2-5)	3 (2-4)	4 (3-5)	<0.01
C-reactive protein (mg/L)	97 (58-160)	97 (57-143)	150 (65-198)	0.19
Procalcitonin (µg/L)	0.18 (0.06-0.48)	0.13 (0.06-0.48)	0.24 (0.13-0.86)	0.16
D-dimer (µg/L)	323 (171-670)	295 (166-641)	374 (301-1167)	0.11
Leukocyte count ($x10^9$	7.58 (4.84-	7.53 (4.48-	7.78 (6.88-	0.36

cells/L)	10.57)	10.57)	9.97)	
Lymphocyte count (x10^9 cells/L)	0.80 (0.55-1.11)	0.76 (0.50-1.07)	0.96 (0.65-1.36)	0.08
IL-6 (pg/mL)	55 (31-148)	52 (30-121)	163 (64-307)	<0.01
PaO2/FiO2 (mmHg)	118 (90-160)	123 (91-161)	110 (78-119)	0.16
PaCO2 (mmHg)	35 (31-38)	35 (31-38)	35 (31-35)	0.79
First CARE score	9 (6-14)	10 (5-15)	8 (7-12)	0.75
Second CARE score	8 (4-14)	8 (4-14)	8 (6-13)	0.99
Delta CARE score	-1 (-5-3)	-1 (-5-3)	-3 (-6-5)	0.87
Endotracheal intubation (n [%])	83 (58)	64 (52)	19 (95)	<0.01
Duration of invasive mechanical ventilation (days)	8 (6-13)	7 (4-11)	17 (11-25)	0.18
Pronation (n [%])	85 (60)	71 (58)	14 (70)	0.05

Data are reported as median (interquartile range) or number (percentage), as appropriate. The Wilcoxon's rank-sum test and the Fisher's exact test were applied, as appropriate.

The delta CARE score is the difference between the first and the second CARE score.

Abbreviations: SOFA, sequential organ failure assessment; IL-6, interleukin-6; PaO2/FiO2, arterial partial pressure of oxygen to inspired oxygen fraction ratio; PaCO2, arterial partial pressure of carbon dioxide.
