# Supplementary material for: Chest X-ray Does Not Predict the Risk of Endotracheal Intubation and Escalation of Treatment in COVID-19 Patients Requiring Noninvasive Respiratory Support
Source: J Clin Med. 2022 Mar 16;11(6):1636. doi: 10.3390/jcm11061636 (PMC8950017; doi:10.3390/jcm11061636)
Supplement: Supplementary file 1 [file jcm-11-01636-s001.zip › Table S6.pdf]

Table S6. Logistic regression for hospital mortality

Variable	Univariable		Multivariable	
	OR (95% CI)	p-value	OR (95% CI)	p-value
First CARE score	0.98 (0.91-1.05)	0.55		
Age	1.09 (1.03-1.15)	<0.01	1.14 (1.03-1.26)	0.01
Female gender	0.53 (0.17-1.68)	0.28		
Days since symptoms onset	1.03 (0.92-1.16)	0.59		
SOFA score	1.42 (1.09-1.84)	0.01	1.29 (0.91-1.84)	0.15
Charlson comorbidity index	1.19 (1.01-1.40)	0.04	1.09 (0.78-1.51)	0.62
C-reactive protein	1.00 (1.00-1.01)	0.12		
Procalcitonin	0.99 (0.85-1.15)	0.88		
D-dimer	1.00 (1.00-1.00)	0.89		
Leukocyte count	1.06 (0.97-1.15)	0.18		
Lymphocyte count	0.99 (0.74-1.33)	0.95		
IL-6	1.00 (1.00-1.00)	0.08		
PaO2/FiO2	0.99 (0.98-1.00)	0.17		
PaCO2	1.05 (1.00-1.11)	0.07		

Endotracheal intubation	17.50 (2.27-135.00)	<0.01	33.50 (3.49-122.00)	<0.01
Duration of invasive mechanical ventilation	1.09 (0.99-1.20)	0.08		
Pronation	4.20 (0.91-19.40)	0.07		

Abbreviations: OR, odds ratio; CI, confidence interval; SOFA, sequential organ failure assessment; IL6, interleukin-6; PaO2/FiO2, arterial partial pressure of oxygen to inspired oxygen fraction ratio; PaCO2, arterial partial pressure of carbon dioxide.

The variance inflation factors were 1.43 for age, 1.01 for the SOFA score, 1.49 for the Charlson comorbidity index, and 1.15 for endotracheal intubation.
